# Efficacy and safety of epidural steroid injection following discectomy for patients with lumbar disc herniation: A protocol

**DOI:** 10.1097/MD.0000000000021220

**Published:** 2020-07-17

**Authors:** Jianping Cai, Wei Jiang, Beiming Qiu, Yuguang Song

**Affiliations:** Department of Orthopedics II, The No.2 People's Hospital of Yibin, Sichuan Province, China.

**Keywords:** epidural steroid injection, lumbar disc herniation, lumbar microdiscectomy, study protocol

## Abstract

**Background:**

Concerns exist regarding the analgesia effect and safety of epidural steroid injection (ESI) after discectomy. There is an urgent need of studies that efficiently control for confounding, conduct comprehensive and consecutive observation of potential risks of ESI, and investigate its clinical applicability. We thus further designed a randomized controlled study to assess the efficacy of ESI on postoperative pain and complications in patients undergoing unilateral lumbar microdiscectomy.

**Methods:**

This prospective, blinded randomized controlled trial was conducted at our single hospital. This study was approved by the Committee at the No.2 People's Hospital of Yibin. All procedures were performed by a single surgeon and informed consent was obtained from each patient. Ninty eligible patients diagnosed at our institution with herniated lumbar disc during a period from June 2020 to July 2021 will be assessed. Group 1 was a mixture of 1 mL of 0.5% bupivacaine and 10 mg of triamcinolone acetonide in 1 mL. Group 2 was a mixture of 1 mL of 0.5% bupivacaine and 1 mL of normal saline. The primary outcome measure was the amount of morphine consumption from a patient-controlled analgesia pump at 12, 24, and 48 hours after surgery. The following secondary outcomes were also assessed: postoperative pain score, back pain score, functional disability, and adverse effect.

**Conclusions:**

We hypothesized that the ESI was associated with lower pain score, morphine consumption, and hospital stay, with no significant difference in complications for ESI application after lumbar discectomy in lumbar disc herniation when compared with placebo.

**Trial registration:**

This study protocol was registered in Research Registry (researchregistry5683).

## 1 Introduction

Lumbar disc herniation is considered to be one of the main causes of sciatica, and lumbar discectomy is the most popular surgical procedure performed in patients with sciatica in China.^[1]^ Discectomy significantly relieves back pain as well as radicular symptoms after the operation. However, many patients with lumbar discectomy experience moderate to severe back pain and radicular leg pain. Lumbar discectomy will remove the mechanical cause of pain, but the painful inflammatory process might persist for a few more days. In addition, the inflammation together with the surgical intervention might negatively influence the long-term results by initiating fibrosis which is considered one of the reasons for late reappearance of pain.^[2–4]^

Reduction of inflammation and edema of the affected nerve root should, theoretically, reduce the postoperative pain intensity. Epidural steroid injection (ESI) is widely used to treat pain originating from the spine, such as pain accompanying spinal stenosis, intervertebral disc herniation, and other degenerative spinal pathologies.^[5–8]^ Many surgeons routinely use ESI during lumbar discectomy to reduce traumatic nerve root inflammation and edema. However, glucocorticoids have multiple adverse effects. Administration of exogenous glucocorticoids dramatically reduces bone mineral density and increases the risk of fracture.^[9–13]^

Previous studies on the effect of ESI following discectomy for herniated lumbar disc disease has been inconclusive and without long-term follow-up.^[14–16]^ Other studies on perioperative steroid vary within administration, diagnostic criteria, surgical technique, and postoperative care.^[17]^ Furthermore, the number of patients in most studies has been low and 2 randomized controlled studies (RCTs) showed no significant effect. However, the other studies reported a positive effect of steroids on pain. Four previous meta-analyses were published^[18–21]^; 2 meta-analyses reported that intraoperative ESI when compared to placebo was effective in reducing pain in the early stage and reducing consumption of analgesia with no statistically significant differences in terms of overall complications and infection in lumbar discectomy.^[18,19]^ One meta-analysis reported that intraoperative ESI application offers some benefits in pain control with a significant reduction in the length of hospital stay.^[20]^ The most recent meta-analysis indicated a lower back and leg pain score, decreased morphine consumption, and shorter hospital stay without difference in complications for ESI application compared to placebo after lumbar discectomy in lumbar disc herniation that was not detected in previous meta-analyses.^[21]^ However, these meta-analyses still have many limitations.

Despite the evidence above, concerns exist regarding the analgesia effect and safety of ESI after discectomy. There is an urgent need of studies that efficiently control for confounding, conduct comprehensive and consecutive observation of potential risks of ESI, and investigate its clinical applicability. Considering different conclusion and limitation of previous studies, we thus further designed a RCT to assess the efficacy of ESI on postoperative pain and complications in patients undergoing unilateral lumbar microdiscectomy. We hypothesized that the ESI was associated with lower pain score, morphine consumption, and hospital stay, with no significant difference in complications for ESI application after lumbar discectomy in lumbar disc herniation when compared with placebo.

## 2 Material and method

### 2.1 Study design

This prospective, blinded randomized controlled trial was conducted at our single hospital. This study was approved by the Committee at the No.2 People's Hospital of Yibin (YBH22470), and the protocol of the study was registered in Research Registry (researchregistry5683). All procedures were performed by a single surgeon and informed consent was obtained from each patient. Ninty eligible patients diagnosed at our institution with herniated lumbar disc during a period from June 2020 to July 2021 will be assessed.

Eligible patients were those undergoing primary single-level unilateral lumbar discectomy. All patients involved in the study had been diagnosed with a prolapsed intervertebral lumbar disc with nerve compression demonstrated on magnetic resonance imaging, with clinically correlated clinical findings and had failed conservative treatment before enrolment. Excluded were patients with central or lateral spinal stenosis due to spondylosis or disc degeneration who needed bilateral decompression, laminectomy, or fusion, and patients with cauda equina syndrome who needed acute operative treatment.

### 2.2 Randomization

**Figure 1:**
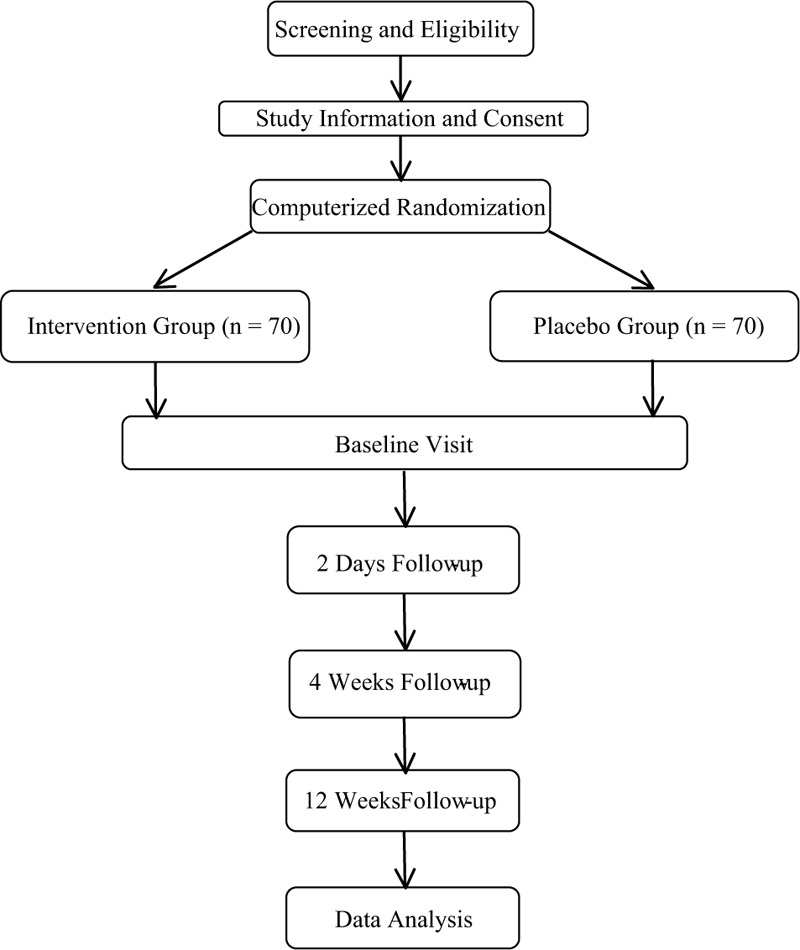
Consolidated Standards of Reporting Trials Statement flow diagram.

The patients were randomly allocated by stratified block randomization using a computer-generated random number into the treatment group or the placebo group. The allocation was made by opening sequentially numbered opaque sealed envelopes. Concealment of allocation was maintained until the time of the study drugs or saline was injected. All other clinical personnel, participants, and outcome assessors were blinded to the intervention. After discharge, the participant's personal information was eliminated from the study number and was therefore not traceable back to the patients (Fig. 1).

### 2.3 Interventions

Group 1 was a mixture of 1 mL of 0.5% bupivacaine and 10 mg of triamcinolone acetonide in 1 mL. Group 2 was a mixture of 1 mL of 0.5% bupivacaine and 1 mL of normal saline. The trained assessor and the patient were blinded to treatment assignment for the duration of the study. Study and data monitor was not blind.

In both groups, prophylactic cephalosporin 1500 mg intravenous was given 15 minutes before surgery. General anesthesia was standardized with remifentanil, propofol, rocuronium, glycopyron, and at the end of operation fentanyl 100 μg and toradol 30 mg. For treatment of postoperative nausea and vomiting dihydrobenzperidol 0.625 to 125 mg intravenous was given at induction of anesthesia and ondansetron 4 mg intravenous at the end of anesthesia. Both groups received postoperative pain treatment with acetaminophen 1 g 6 hourly and celecoxib 200 mg daily. When necessary oxycontin 20 mg twice daily, oxynorm 5 mg at demand, or morphine 50 to 100 μg/kg intravenous was given. Nutrition with protein supplements >1.3 g/kg, iron, and vitamins were given from the day before operation including the day of operation and until discharge.

### 2.4 Outcomes and measures

The primary outcome measure was the amount of morphine consumption from a patient-controlled analgesia pump at 12, 24, and 48 hours after surgery. The following secondary outcomes were also assessed: postoperative pain score, back pain score, functional disability, and adverse effect.

Postoperative pain intensity at rest and pain on cough were assessed at 12, 24, and 48 hours after surgery on a verbal numerical rating scale ranging from 0 (no pain) to 10 (worst pain possible). Back pain intensity was assessed before surgery and at 1 week, 1 month, and 3 months after surgery on a verbal numerical rating scale. Functional disability was assessed before surgery and at 1 and 3 months after surgery using the Oswestry Disability Index, a disease-specific questionnaire designed for use in patients with low back pain that evaluates the patient's overall level of functional disability in terms of percentage score, with higher values representing higher levels of disability. General health status was assessed before surgery and at 1 and 3 months after surgery using the Short Form SF-36 Health Survey Questionnaire, a validated general health questionnaire that document functional status, well-being, and patients’ overall evaluation of their health as a percentage score, with 100% representing the best possible function and 0% the worst possible function. Adverse effect such as sedation and nausea/vomiting was assessed during patients using a patient-controlled analgesia pump.

### 2.5 Sample size calculation

The sample size was calculated to detect a mean difference in 24-hour postoperative morphine consumption between ESI and placebo after unilateral lumbar microdiscectomy. For the previous meta-analysis, the mean and standard deviation of 24-hour postoperative morphine consumption in ESI group were 31.3 and 3.62, respectively. From this, it was determined that 50 subjects would be required to reach an α value of 0.05 and a power of 85%. It was estimated that the attrition rate due to canceled surgery or reasons of late patient ineligibility could be up to 20% and, therefore, to account for this, the final sample size selected was n = 140 (70 per group).

### 2.6 Statistical analysis

The statistical analyses in this study were performed using the Statistical Package for the Social Sciences 20.0 software (SPSS Inc, Chicago, IL). Continuous variables were presented in the form of mean ± standard deviation or error. The Kolmogorov-Smirnov normality test was used to assess continuous variables. Group comparisons on the variables that showed normal distribution were performed using 1-way analysis of variance. Mann-Whitney *U* variance analysis was used for discrete numerical variables that did not show normal distribution. Relationships between the categorical variables were determined by preparing crosstabs and using the χ^2^ test. *P* < .05 was accepted as statistically significant.

## 3 Discussion

Steroids suppress the production of prostaglandins by inhibiting Cyclooxygenase-2 mRNA expression centrally and peripherally. Furthermore, steroids inhibit mediators of inflammatory hyperalgesia, such as tumor necrosis factor-α, interleukin-1β, and interleukin-6. These effects require protein synthesis and have a latency of onset. Another late effect of steroids is suppression of proliferation of intercellular proteoglycans and collagen, thereby reducing adhesion and formation of fibrosis.^[22,23]^ Recently, a shorter onset time for steroid effect has been found. The mechanism of this action is probably transmitted by cell membrane receptors decreasing release of glutamate and γ-amino butyric acid.^[24]^ Based on these action mechanisms, the effects of ESI administration after a lumbar discectomy have been studied extensively. However, the results have previously been inconclusive. Therefore, we further conducted a double-blinded RCT to assess the efficacy of ESI on postoperative pain and complications in patients undergoing unilateral lumbar microdiscectomy. The strength of this study was its prospective and randomized design.

## Author contributions

**Conceptualization:** Jianping Cai.

**Data curation:** Jianping Cai.

**Formal analysis:** Jianping Cai, Wei Jiang.

**Funding acquisition:** Yuguang Song.

**Investigation:** Jianping Cai, Wei Jiang.

**Methodology:** Jianping Cai, Wei Jiang.

**Resources:** Yuguang Song.

**Software:** Jianping Cai, Wei Jiang.

**Supervision:** Yuguang Song.

**Validation:** Beiming Qiu.

**Visualization:** Beiming Qiu.

**Writing – original draft:** Jianping Cai.

**Writing – review & editing:** Yuguang Song, Beiming Qiu.
